# Using machine learning methods for supporting GR2M model in runoff estimation in an ungauged basin

**DOI:** 10.1038/s41598-021-99164-5

**Published:** 2021-10-07

**Authors:** Pakorn Ditthakit, Sirimon Pinthong, Nureehan Salaeh, Fadilah Binnui, Laksanara Khwanchum, Quoc Bao Pham

**Affiliations:** 1 School of Engineering and Technology, Walailak University, Nakhon Si Thammarat, 80161 Thailand; 2 School of Languages and General Education, Walailak University, Nakhon Si Thammarat, 80161 Thailand; 3 Center of Excellence in Sustainable Disaster Management, Walailak University, Nakhon Si Thammarat, 80161 Thailand; 4 Institute of Applied Technology, Thu Dau Mot University, Thu Dau Mot City, Binh Duong Province 821389 Vietnam

**Keywords:** Environmental sciences, Hydrology

## Abstract

Estimating monthly runoff variation, especially in ungauged basins, is inevitable for water resource planning and management. The present study aimed to evaluate the regionalization methods for determining regional parameters of the rainfall-runoff model (i.e., GR2M model). Two regionalization methods (i.e., regression-based methods and distance-based methods) were investigated in this study. Three regression-based methods were selected including Multiple Linear Regression (MLR), Random Forest (RF), and M5 Model Tree (M5), and two distance-based methods included Spatial Proximity Approach and Physical Similarity Approach (PSA). Hydrological data and the basin's physical attributes were analyzed from 37 runoff stations in Thailand's southern basin. The results showed that using hydrological data for estimating the GR2M model parameters is better than using the basin's physical attributes. RF had the most accuracy in estimating regional GR2M model’s parameters by giving the lowest error, followed by M5, MLR, SPA, and PSA. Such regional parameters were then applied in estimating monthly runoff using the GR2M model. Then, their performance was evaluated using three performance criteria, i.e., Nash–Sutcliffe Efficiency (NSE), Correlation Coefficient (r), and Overall Index (OI). The regionalized monthly runoff with RF performed the best, followed by SPA, M5, MLR, and PSA. The Taylor diagram was also used to graphically evaluate the obtained results, which indicated that RF provided the products closest to GR2M's results, followed by SPA, M5, PSA, and MLR. Our finding revealed the applicability of machine learning for estimating monthly runoff in the ungauged basins. However, the SPA would be recommended in areas where lacking the basin's physical attributes and hydrological information.

## Introduction

Precisely estimating hydrological parameters in the ungauged basin has drawn the attention of hydrologists and water resources engineering^1^. In meteorology, the assessment of runoff is extremely important^2^, especially in areas where there is no measuring station that cannot be calibrated. Therefore, the regionalization method is optional for transferring model parameters from the gauged basin to the ungauged basin^3,4^. The popular regionalization methods are physical similarity, spatial proximity, and regression^5^. Previously, many studies have been conducted and compared the performance of the regionalization methods to predict total streamflow or direct runoff^6^ in the ungauged catchment with various hydrological models (WASMOD^7^, VIC^8^, SWAT^9^, GR4J^10^, HMETS^10^, MOHYSE^10^, and HEC-HMS^11^) for different regions. Some of them showed that distance-based (spatial proximity, physical similarity) outperformed regression methods^7,8^. The combined watershed classification of inverse distance weighted (IDW) and physical similarity methods was investigated to predict streamflow in the ungauged catchment by Kanishka and Eldho^9^. Swain and Patra^3^ pointed that the spatial proximity between the gauged catchment and the ungauged catchment gave better results than the physical similarity for predicting the continuous streamflow. Arsenault et al.^10^ studied the efficacy of three regionalization methods: multiple linear regression (MLR), spatial proximity, and physical similarity, to predict current flow in ungauged catchments of Mexico. They showed that transferring a set of parameters from a nearby reservoir is the most efficient method for estimating the runoff in ungauged basins. Tegegne and Kim^12^ proposed a catchment runoff-response similarity (CRRS) method to identify crucial properties for supporting the hydrological similarity. This approach was conducted with South Korea's Geum River Basin (GRB) and Ethiopia's Lake Tana Basin (LTB). The results showed that CRRS performed better than the others. Koçyiğit et al.^13^ found that when the geometric dimensions of the sub-basin changed, the hydrological parameters of those sub-basins also changed.

Recently, machine learning has been popular and widely applied in hydrology and water resources engineering. Hussain and Khan^14^ indicated that random forest (RF) was more effective than multilayer perceptron (MLP) and support vector regression (SVR) for monthly flow forecasting in Hunza River, Pakistan. Schoppa et al.^15^ showed that random forest could simulate both small and medium floods equivalent to the HYDROMAD model. Wang and Wang^16^ found that multiple linear regression (MLR) and M5P model tree (M5P) gave more precisely in predicting the daily water level in Lake Erie than Gaussian process (GP), multilayer perceptron (MLP), random forest (RF), and k-nearest neighbor (KNN). For predicting the flow in ungauged basins, Araza et al.^17^ applied the regionalizing RF models in the 21 mountainous regions of Luzon, Philippines. However, very few researches have been conducted for applying machine learning for estimating hydrological model parameters. Recently, Saadi et al.^18^ explored RF algorithms' ability to express the relationship between hourly hydrological model (GR4H) parameters and climate/landscape catchment in the 870 catchments in the United States and 1355 catchments in France.

The GR2M model (Rural Genius model) has lately been used to simulate a watershed's hydrological features due to climate variability on hydrologic regimes^19^ and evaluate the effects of climate change on runoff^20^. It was utilized to screen hydrologic data to study hydrological response across the Lower Mekong Basin^21^. Boulariah et al.^22^ found that the GR2M model gave better performance than the ABCD. The runoff simulation under various climate conditions in the Wimmera catchment was studied using four monthly rainfall-runoff models: abcd, Budyko, GR2M, and WASMOD by Topalović et al.^23^. The effects of climate scenarios on monthly river runoff in the Cheliff, Tafna, and Macta in North-West^24^ were conducted by the GR2M model. Rintis and Setyoasri^25^ found that the GR2M model gave a comparable performance to Mock and NRECA methods. The GR2M model and Artificial Neural Network were utilized to reconstruct monthly river flow for Irish catchments^26^. The regionalized GR2M model’s parameters were developed to predict monthly runoff in the ungauged basins for northern Algeria^27^.

Thailand's southern region, located in the tropical climate, has been experiencing water-related disasters (e.g., flooding and drought). The comprehension of spatiotemporal hydrological characteristics, especially runoff, can reduce or alleviate extensive damage to human lives and properties and sustain economic growth^28–31^. As mentioned earlier, accuracy in estimating monthly runoff variation in the ungauged basin is vital for water resources planning and management. The regionalization approach has been widely accepted for this purpose. However, in Thailand, especially in the southern region, there was no research work using machine learning to estimate the regionalized GR2M parameters. To fulfill this gap in the literatures, our research aimed to investigate and compare the performance of two main regionalization approaches, i.e., regression-based and distance-based methods, to determine the GR2M model parameters for monthly runoff estimation at an ungauged basin in southern Thailand. It is the first attempt to discover the most practical approach for obtaining the regionalized GR2M parameters under the data scarcity context in the south region, Thailand. The following is an outline of how this article is structured: (1) the rationale for conducting this research work and its related literature review; (2) the study area explanation; (3) The GR2M description; (4) the framework and its detailed information of this research methodology; (5) our results, findings, and discussion; (6) this research conclusion and its contributions.

### Study area

**Figure 1:**
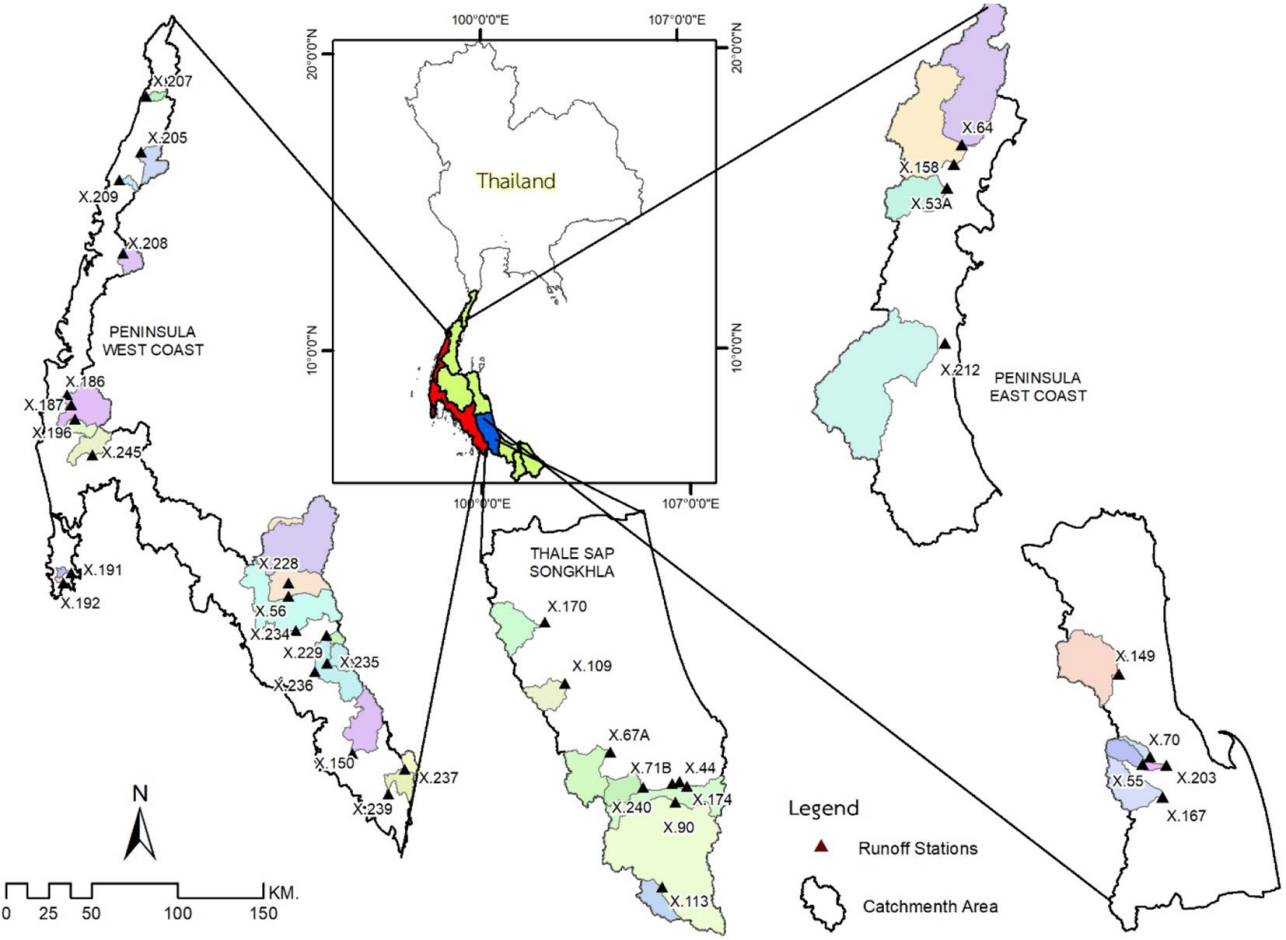
The station locations of rainfall, runoff, and weather stations of the study area. (This map was created by QGIS version 3.12, which can be accessed on https://qgis.org/en/site/).

Our research work focused on three of five major river basins in the southern basin of Thailand (see Fig. 1): the Peninsula-East Coast (26,024 km^2^), Peninsula-West Coast (18,841 km^2^), and Thale Sap Songkhla (8484 km^2^) due to the available hydrological information. This peninsula area is located in between the Andaman Sea and the South China Sea. In the northern and central regions, there is a long western mountain range, and in the midst of the ridge's southern region is the Nakhon Si Thammarat ridge. The monsoon winds from the northeast and southwest are primarily responsible for its climatological characteristics. A minor coastal plain exists in the Peninsula-East Coast watershed, with short rivers of fewer than 150 km draining into the Gulf of Thailand. This watershed had nine runoff stations used for data analysis. The Peninsula-West Coast Watershed features short rivers that run into the Andaman Sea in the west and southwest. The runoff information was collected and analyzed from nineteen runoff stations. Thale Sap Songkhla watershed mainly locates in Songkhla, Phatthalung, and the lower part of Nakhon Si Thammarat. There were nine runoff stations available in the Thale Sap Songkhla watershed.

### GR2M model

**Figure 2:**
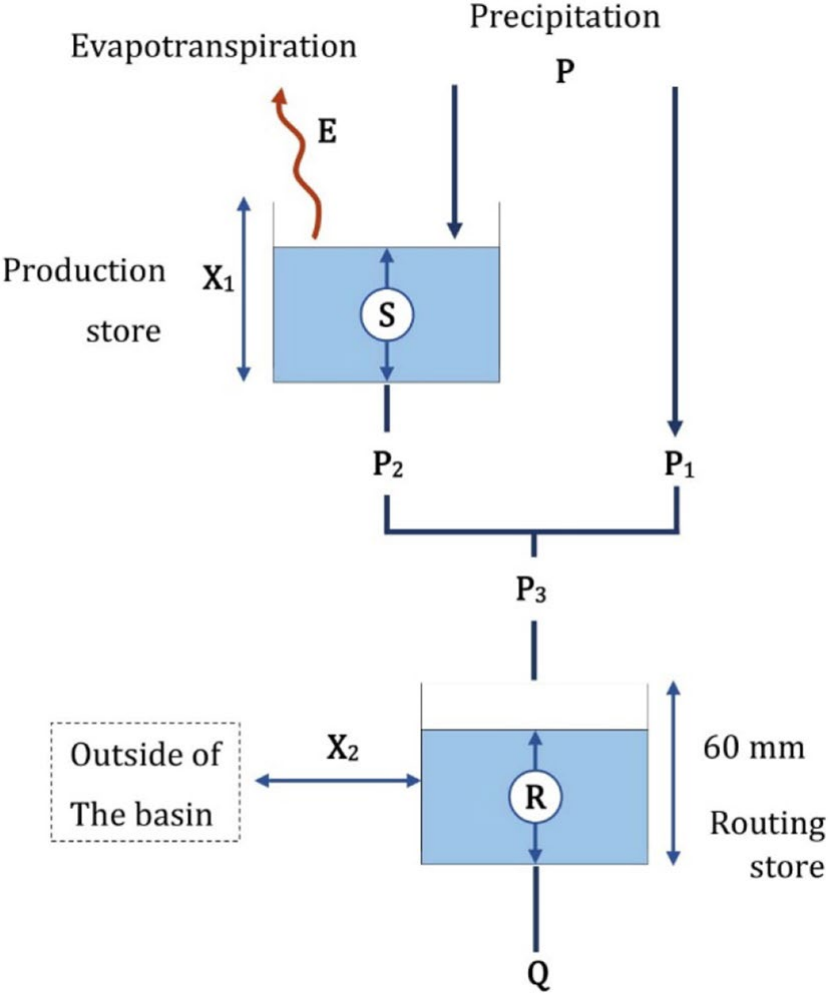
The GR2M model working process. *Source*: Adapted from Bachir et al.^38^, Rwasoka et al.^39^.

The GR2M is a conceptual monthly rainfall-runoff mathematical model developed by Demagref in the late 1980s. Later on, several versions have been continuously being improved its efficiency by Kabouya^32^, Makhlouf^33^, Mouelhi^34^ until Mouelhi et al.^35^. This study used the version of 2006b. Literature reviews showed its performance, applicability, and simplicity compared to other models^36^ due to requiring two parameters: the ability to keep moisture in the soil (X_1_) and the water exchange coefficient (X_2_). Monthly rainfall, runoff, and evapotranspiration are the only three meteorological and hydrological data necessary^35,37^. Total runoff hydrograph, soil moisture content, groundwater flow, etc., are the model results. The water balance concept with two reservoirs was utilized for the GR2M model, as presented in Fig. 2. In the upper reservoir, the basin’s soil moisture (S) depends on production store: X_1_ (mm). And the lower reservoir is river flow (R), which is regulated by the exchange coefficient water: X_2_. and a maximum capacity of 60 mm. Starting with precipitation penetrated the soil, soil moisture is at the level: S1 (mm). When the soil has reached saturation, the rainfall excess occurs P1 (mm). Some soil moisture can decrease during that process due to evapotranspiration E, resulting in the soil moisture remains at level: S2 (mm). Some soil water infiltrates into the soil as subsurface water: P_2_ (mm) and conglomerate with rainfall excess to be surface runoff: P_3_ (mm). The surface runoff flows into the river combining with the rest water from the previous month: R (mm). The river runoff can change depending on the direction of water flowing into or out from the basin. Finally, the total runoff hydrograph is obtained.

### Research methodology

**Figure 3:**
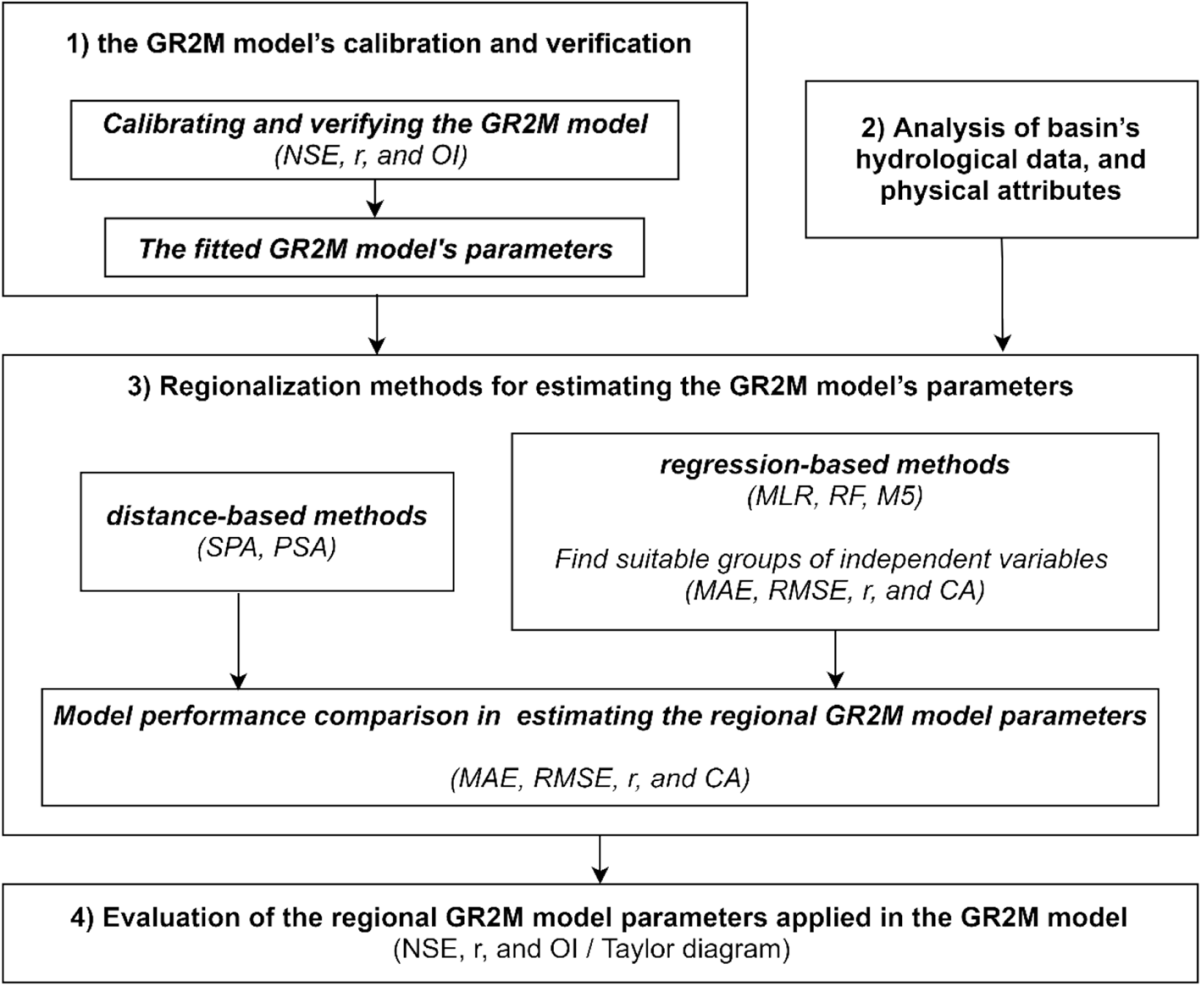
Framework for research methodology.

In this study, the research methodology (Fig. 3) consisted of four main steps, i.e., (1) the GR2M model’s calibration and verification; (2) analysis of basin’s hydrological data and physical attributes; (3) regionalization methods for estimating the GR2M model’s parameters and their performance comparison; and (4) performance evaluation of the regional GR2M model parameters in estimating monthly runoff. The detailed information for each step can be explained as the following.

### The GR2M model’s calibration and verification

The model’s calibration and verification were conducted to make the GR2M model reliable for estimating monthly runoff for 37 different runoff stations in the Southern Basins, Thailand. Before calibrating and verifying the GR2M model, it requires a warm-up period to determine the suitable initial values of X_1_ and X_2_ so that the model can imitate the existing hydrological characteristics of the considering basin. For doing this, the initial R value raining from 10 to 60 mm was sought. And the appropriate warm-up periods of 4 to 7 months were discovered, depending on the runoff station characteristics. In this study, the fitted values of X1 and X2 parameters for each runoff station were automatically determined with Microsoft Excel Solver's help by setting root mean square error (RMSE) as an objective function and the constraints of X1 and X2 parameters. The available monthly rainfall, evapotranspiration, and runoff data for each runoff station ranged from 41 to 80 months, resulting in the calibration and verification periods were 22 to 48 months and 10 to 39 months, respectively.

### Analysis of basin’s hydrological data, and physical attributes

**Figure 4:**
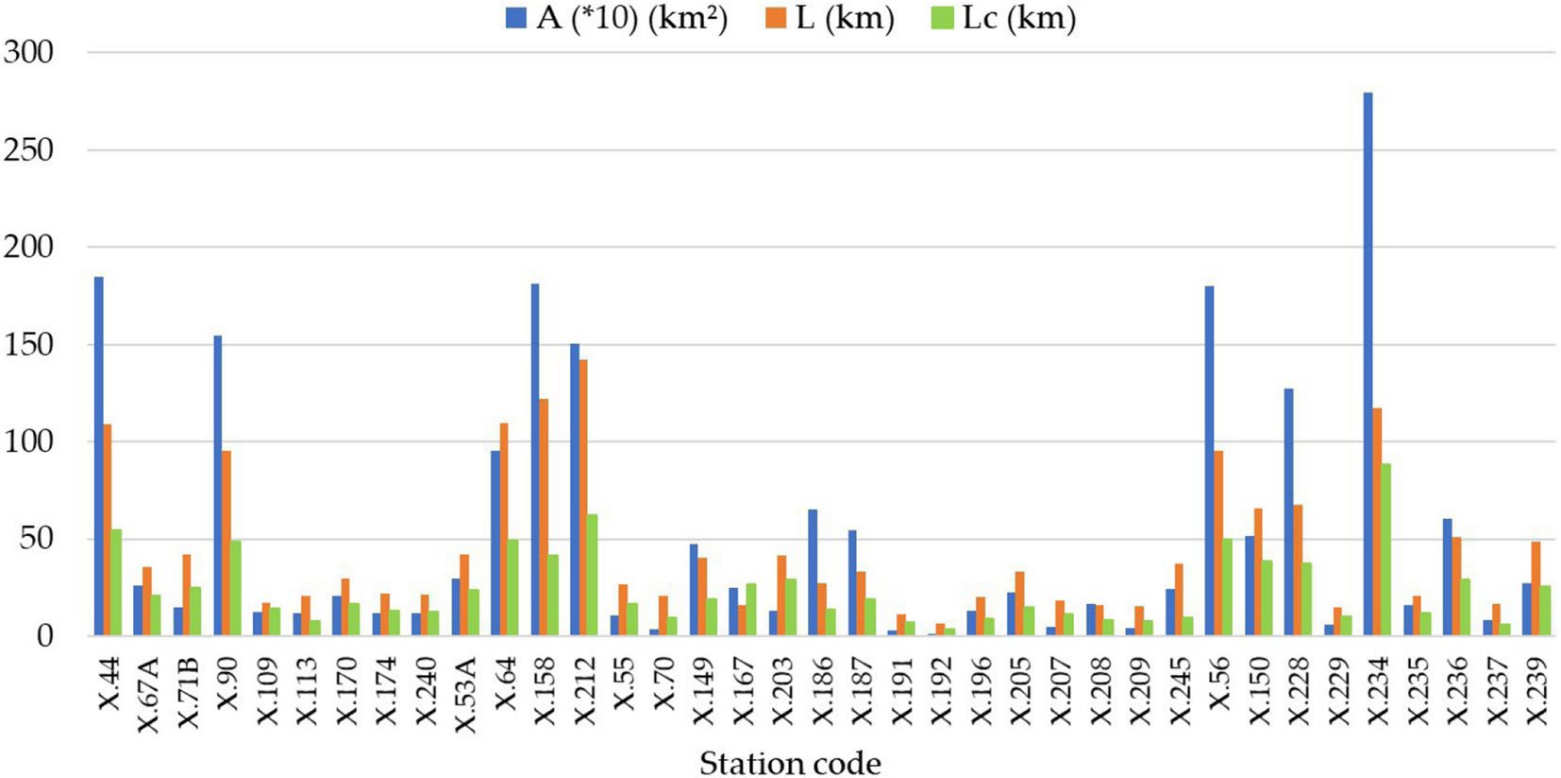
Physical characteristics information of the 37 runoff gauged stations.

We collected the monthly runoff (37 stations), rainfall (38 stations) and air temperature (13 stations) information from the Royal Irrigation Department (RID) and the Thai Meteorological Department (TMD). Figure 2 depicts the locations of rainfall, runoff, and weather. Areal rainfall and air temperature information for each runoff gauged station was analyzed by using Thiessen polygon. Table 1 shows the summary statistical values of hydrological data and physical characteristics of runoff gauged station used in this analysis. We used Thornthwaite^40^ equation to calculate monthly evapotranspiration. Figure 4 shows the physical characteristics information of the 37 runoff gauged stations, including basin area (A), river length (L), and river length from the basin’s centroid to the basin outlet (L_c_) of runoff gauged station, were determined with the help of QGIS, a free and open-source geographic information system software. We examined the time matching to choose the appropriate times for calibrating and verifying the model. Hence, all periods for running the GR2M model were in the range of 41 to 80 months. And its calibration and verification periods were in the range of 22 to 49 months and 10 to 39 months, respectively.

**Table 1:** Summary statistical values of hydrological data and physical characteristics of runoff gauged station used in this analysis.

Statistical value	Runoff (mm)	Rainfall (mm)	ET (mm)
**Hydrological data**			
Maximum	1615.43	1562.30	248.95
Minimum	289.45	0.00	94.37
Average	124.41	197.33	148.86
Standard deviation	119.29	155.28	19.31

Statistical value	A (km^2^)	L (m)	L_c_ (m)
**Physical characteristics**			
Maximum	2797.96	141,856.48	88,246.54
Minimum	12.89	6311.62	3711.12
Average	532.70	45,044.77	24,036.15
Standard deviation	680.71	36,572.41	18,911.97

### Regionalization methods for estimating the GR2M model’s parameters

Estimating the GR2M model’s parameters in the ungauged basin was analyzed by using the regionalization concept. It is a method that transfers model parameters from donor catchments to the target station or ungauged catchments^41^. In this study, two regionalization methods, i.e., regression-based methods, and distance-based methods, were investigated and compared their performance.

#### Regression-based methods

Regression analysis was used to determine the relationship between the fitted GR2M model parameters and three basin's physical characteristics (A, L, and L_c_), and thirteen hydrological data, including monthly average areal rainfall for 12 months, and annual average areal rainfall. Each fitted GR2M model parameter (i.e., X_1_ and X_2_) was a dependent variable, while the basin's physical characteristics and hydrological data were independent variables. We conducted three scenarios for selecting the most suitable group of independent variables, that is, (1) using only the basin’s physical characteristics, (2) using only hydrological data, and (3) combining those mentioned variables in the both scenarios 1 and 2. Three regression-based methods were selected herein, Multiple Linear Regression (MLR), Random forest (RF), and M5 Model Tree (M5). The last two methods are based on a data-driven model.

### Multiple linear regression analysis (MLR)

1 $${\text{y}} = {\text{a}}_{1} {\text{x}}_{1} + {\text{a}}_{2} {\text{x}}_{2} + \cdots + {\text{a}}_{{{\text{n}} - 1}} {\text{x}}_{{{\text{n}} - 1}} + {\text{a}}_{{\text{n}}} {\text{x}}_{{\text{n}}} + {\text{b}}$$

where y is the dependent variable, x_i_ is the independent variable; a_i_ is regression coefficient, b is constant of regression equation, and n is number of the independent variable. We utilized regression function in Microsoft excel to develop regionalized GR2M model parameter equations.

### Random forest (RF)

Random forest (RF) popular modification of decision trees and one of the ensemble techniques, was first introduced by Breiman in early 2001^42^. It can use for data classification and regression. The advantage of RF is that it can find a series of complex relationships between predictors and responses without any relationships between them by including decision trees^42^.RF creates several trees based on decision trees method, where every tree is produced by arbitrarily selecting training data set, called bagging process, and attributes (or features) from the input vector. By the voting method from the predictive output of every tree created, the model prediction is finally obtained. In regression, the tree predictor proceeds on numerical values as arbitrary to class labels used by the random forest classifier^43^. The most frequently used variable selection measures in tree induction are the Information Gain Ratio criterion^44^ and Gini index^45^. Unlike the M5 model tree, full-grown RF trees are not pruned. One of the key advantages of random forest regression over the M5 model tree is that it is more flexible. The speculation error always converges as the number of trees grows, even if the tree isn’t pruned, and overfitting isn’t a concern because of the Strong Law of Large Numbers^43^. We used WEKA, free and open-source software, and all default RF parameters as recommended by WEKA in our study.

### M5 model tree (M5)

Quinlan 1992^44^ irst developed the M5 model tree by employing a divide-and-conquer strategy to establish the relationship between independent and dependent variables. It can be applied to both qualitative (categorical) and quantitative variables. Building M5 involves three stages. The first stage involves the development of a decision tree by dividing the data set into subsets (or leaves). Second, to prevent an overfitted structure or weak generalizer, the overgrown tree is pruned, and linear regression functions are used to replace the pruned sub-trees. the overgrown tree is pruned and the pruned sub-trees are replaced by linear regression functions. The pruning method requires the merger of some of the lower sub-trees into one node. Finally, the smoothing process is used to compensate for the strong discontinuities that would undoubtedly exist between neighboring linear models on the trimmed trees' leaves, especially for some models with a small number of training samples. For regression-based methods, the suitable group of independent variables for determining two GR2M parameters was investigated. Thus, there were three scenarios, that is, (1) scenario-1: using only the basin’s physical characteristics, (2) scenario-2: using only hydrological information, and (3) scenario-3: combining those mentioned variables in scenarios 1 and 2.

### Distance-based methods

Distance-based methods are a method for determination hydrological model parameters in the ungauged basin by transferring their values from donor catchments to the target station or ungauged catchments. Two approaches are popular recommended: Inverse Distance Weighted (IDW), and Inverse Similarity Weighted (ISW). The IDW value depends on the proximity of the distance, whereas ISW value depends on the similarity of the physical characteristics^7^. By applying IDW and ISW concepts, Spatial Proximity Approach^25^ and Physical Similarity Approach (PSA) were utilized respectively herein and can be concisely explained as follows:

### Spatial proximity approach (SPA)

SPA is the method to select donor stations with a proximity distance to a target station^5^. The distance between a gauged station (or donor station) and ungauged stations (or a target station) can be determined by:

2 $${\text{D}}_{{{\text{ug}}}} = \sqrt {\left( {{\text{x}}_{{\text{u}}} - {\text{x}}_{{\text{g}}} } \right)^{2} + \left( {{\text{y}}_{{\text{u}}} - {\text{y}}_{{\text{g}}} } \right)^{2} }$$

where ${\text{x}}_{{\text{g}}} ,\;{\text{x}}_{{\text{u}}}$ are the latitude (UTM), ${\text{y}}_{{\text{g}}} ,\;{\text{y}}_{{\text{u}}}$ are the longitude (UTM); which g is donor station, and u is the target station, and ${\text{D}}_{{{\text{ug}}}}$ is the distance between g and u stations.

The inverse distance weighted can be calculated as:

3 $${\text{W}}_{{{\text{g}}\_{\text{i}}}} = \frac{{\left( {1/{\text{D}}_{{{\text{ug}}\_{\text{i}}}} } \right)}}{{\sum_{{{\text{i}} = 1}}^{{\text{n}}} \left( {1/{\text{D}}_{{{\text{ug}}\_{\text{i}}}} } \right)}}$$

${\text{W}}_{{{\text{g}}\_{\text{i}}}}$ is the inverse distance weighted, and n is the total number of donor stations.

A parameter of the target station can be obtained by:

4 $${\text{P}}_{{{\text{ug}}}} = \mathop \sum \limits_{{{\text{i}} = 1}}^{{\text{n}}} {\text{W}}_{{{\text{g}}\_{\text{i}}}} {\text{p}}_{{{\text{g}}\_{\text{i}}}}$$

where P_ug_ is the parameter of target station, and p_g_i_ is the parameter of donor station.

#### Physical similarity approach (PSA)

PSA is the method based on the concept that catchments with similar physical characteristics would have similar hydrological behavior^46^.

5 $${\text{SI}}_{{{\text{ug}}}} = \sum_{{{\text{i}} = 1}}^{{\text{k}}} \frac{{\left| {{\text{CD}}_{{{\text{g}},{\text{i}}}} - {\text{CD}}_{{{\text{u}},{\text{i}}}} } \right|}}{{\Delta {\text{CD}}_{{{\text{gi}}}} }}$$

where ${\text{SI}}_{{{\text{ug}}}}$ is the similarity index, ${\text{CD}}_{{{\text{g}},{\text{i}}}} ,{\text{CD}}_{{{\text{u}},{\text{i}}}}$ are the catchment descriptor of donor catchments to the target station; $\Delta {\text{CD}}_{{{\text{gi}}}}$ is the rage of ith catchment descriptor, k is the total number of catchment descriptor.

6 $${\text{W}}_{{{\text{g}}\_{\text{i}}}} = \frac{{\left( {1/{\text{SI}}_{{{\text{ug}}\_{\text{i}}}} } \right)}}{{\sum_{{{\text{i}} = 1}}^{{\text{n}}} \left( {1/{\text{SI}}_{{{\text{ug}}\_{\text{i}}}} } \right)}}$$

${\text{W}}_{{{\text{g}}\_{\text{i}}}}$ (ISW) is the inverse similarity weighted, and n is the total number of donor stations.

7 $${\text{P}}_{{{\text{ug}}}} = \mathop \sum \limits_{{{\text{i}} = 1}}^{{\text{n}}} {\text{W}}_{{{\text{g}}\_{\text{i}}}} {\text{p}}_{{{\text{g}}\_{\text{i}}}}$$

where P_ug_ is the parameter of target station, and p_g_i_ is the parameter of donor station.

Model performance in estimating the regional GR2M model parameters was compared using four statistical indices, including Mean Absolute Error (MAE), Root Mean Squared Error (RMSE), Pearson Correlation Coefficient (r), and Combined Accuracy (CA)^47^.

### Evaluation of the regional GR2M model parameters applied in the GR2M model

Three performance criteria, including Nash–Sutcliffe Efficiency (NSE), Correlation Coefficient (r), and Overall Index (OI), and A Taylor diagram were used for evaluating the applicability of the GR2M Model. The details for each performance criteria can be delineated as the following:

Nash–Sutcliffe Efficiency (NSE) is a prominent index for determining model correctness or model performance, as shown in the following equation:

8 $${\text{NSE}} = 1 - \frac{{\mathop \sum \nolimits_{{{\text{i}} = 1}}^{{\text{n}}} \left( {{\text{Q}}_{{{\text{cal}}}} - {\text{Q}}_{{{\text{obs}}}} } \right)^{2} }}{{\mathop \sum \nolimits_{{{\text{i}} = 1}}^{{\text{n}}} \left( {{\text{Q}}_{{{\text{obs}}}} - \overline{{\text{Q}}}_{{{\text{obs}}}} } \right)^{2} }}$$

The NSE ranges from −  α to 1. If the NSE is near to 1, the observed and calculated runoff are likely to be identical, or it is considered the most efficient or accurate^48^.

The correlation coefficient (r) shows agreement between two variables. The following equation can be used to compute the correlation coefficient between X and Y.

9 $${\text{r}} = \frac{{\mathop \sum \nolimits_{{{\text{i}} = 1}}^{{\text{n}}} \left( {{\text{Q}}_{{{\text{obs}}}} - \overline{{\text{Q}}}_{{{\text{obs}}}} } \right)\left( {{\text{Q}}_{{{\text{cal}}}} - \overline{{\text{Q}}}_{{{\text{cal}}}} } \right)}}{{\sqrt {\mathop \sum \nolimits_{{{\text{i}} = 1}}^{{\text{n}}} \left( {{\text{Q}}_{{{\text{obs}}}} - \overline{{\text{Q}}}_{{{\text{obs}}}} } \right)^{2} } \cdot \sqrt {\mathop \sum \nolimits_{{{\text{i}} = 1}}^{{\text{n}}} \left( {{\text{Q}}_{{{\text{cal}}}} - \overline{{\text{Q}}}_{{{\text{cal}}}} } \right)^{2} } }}$$

The r-value ranges from − 1 to 1. The plus sign (+) indicates the direct relation between observed and predicted values or vice versa^49^.

The overall index (OI) is a model performance criterion that gives the value between − ∝ to 1. The model’s performance is prominent if OI approaches to 1^50^.

10 $${\text{OI}} = \frac{1}{2}\left[ {2 - \frac{{{\text{RMSE}}}}{{{\text{Q}}_{{{\text{obs}},{\text{max}}}} - {\text{Q}}_{{{\text{obs}},{\text{min}}}} }} - \frac{{\mathop \sum \nolimits_{{{\text{i}} = 1}}^{{\text{n}}} \left( {{\text{Q}}_{{{\text{obs}}}} - {\text{Q}}_{{{\text{cal}}}} } \right)^{2} }}{{\mathop \sum \nolimits_{{{\text{i}} = 1}}^{{\text{n}}} \left( {{\text{Q}}_{{{\text{obs}}}} - \overline{{\text{Q}}}_{{{\text{obs}}}} } \right)^{2} }}} \right]$$

where ${\text{Q}}_{{{\text{obs}}}}$ is the observed runoff, ${\text{Q}}_{{{\text{cal}}}}$ is the calculated runoff, ${\overline{\text{Q}}}_{{{\text{obs}}}}$ is the average observed runoff, ${\overline{\text{Q}}}_{{{\text{cal}}}}$ is the average calculated runoff, ${\text{Q}}_{{{\text{obs}},{\text{max}}}}$ is the maximum observed runoff, ${\text{Q}}_{{{\text{obs}},{\text{min}}}}$ is the minimum observed runoff, and n is the number of runoff data.

A Taylor diagram was used herein to comparatively elaborate and evaluate the efficacy among the developed models. This diagram can simultaneously show three statistic parameters, i.e., correlation, root mean square error, and standard deviation.

## Results and discussion

This section presents our finding as follows: (1) calibrating and validating the GR2M Model and its fitted values, (2) the suitable group of independent variables for determining two GR2M parameters using regression-based methods, (3) model performance comparison in estimating the regional GR2M model parameters, and (4) evaluation of the regionalized parameters applied in the GR2M model. The details information is delineated as follows.

### Calibrating and validating the GR2M Model and its fitted values

**Figure 5:**
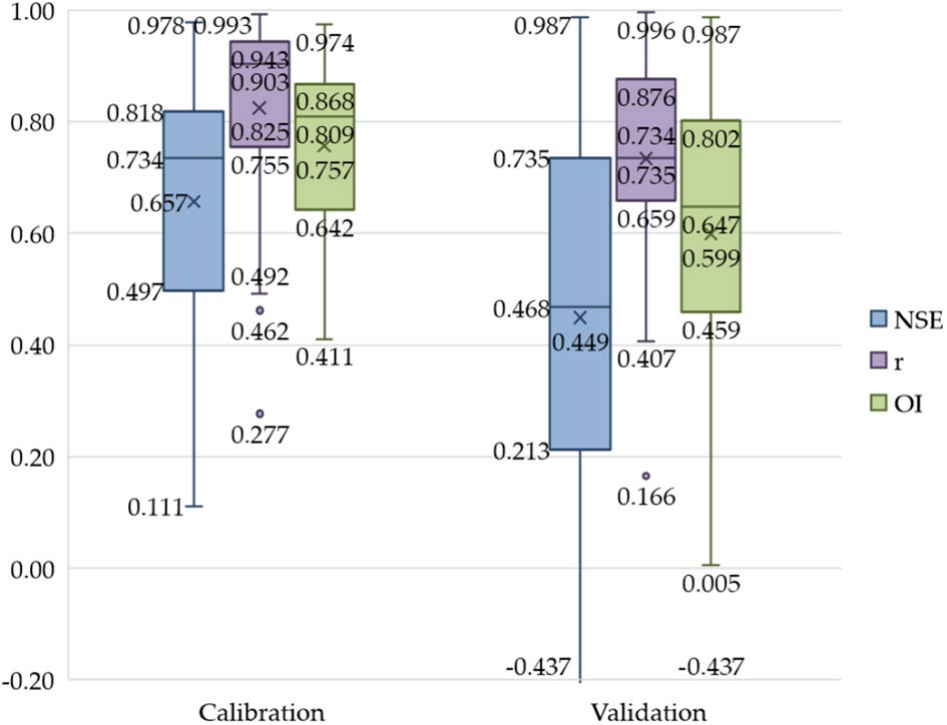
The results of model’s calibration and validation.

**Figure 6:**
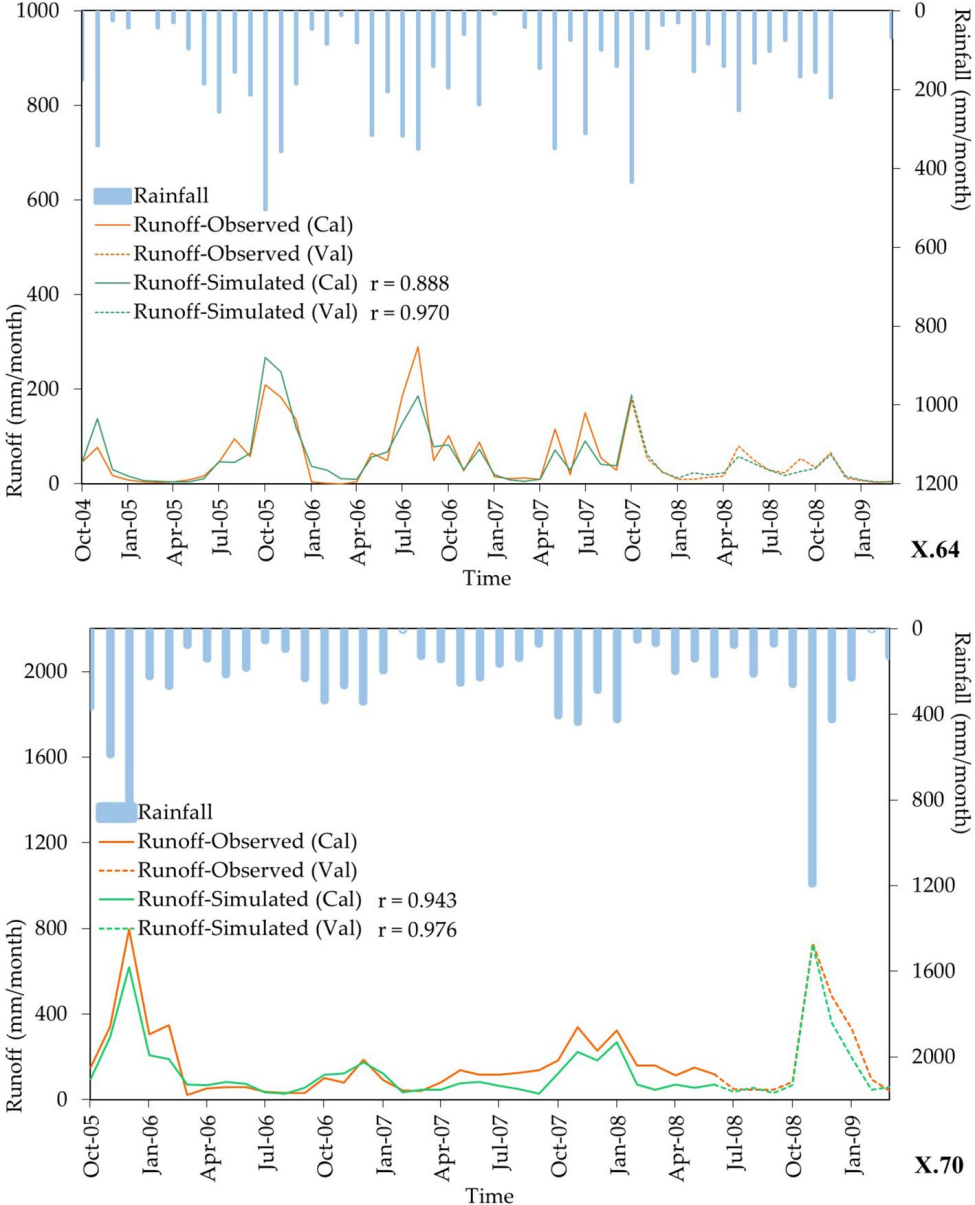
The rainfall and runoff time series at runoff stations X.64 and X.70.

The calibrating and validating results of the GR2M model is depicted as box plot in Fig. 5. The calibrated NSE, r, and OI values were 0.657, 0.825, and 0.757, respectively, and the verified NSE, r, and OI values were 0.449, 0.743 and 0.599, respectively. It was a satisfactory model prediction as suggested by Lian et al.^51^. The obtained r value of more than 0.70 showed a strong positive linear relationship between the calculated and observed runoff data^52^. The OI value of more than 0.60 showing the model had a relatively high accurate prediction. Figure 6 shows two examples of rainfall and runoff time series at the X.64 and X.70 stations, which were obtained from the GR2M model. The monthly rainfall time-series data is shown as a bar chart in blue. The observed and calculated runoff time-series data are shown with the line graphs in orange and green, respectively. And the solid and dot lines indicate the calibrated and validated periods, respectively. The agreement between observed and calculated runoff time-series data with a bit of underestimating the calculated runoff is found.

The statistical values of the fitted GR2M model parameters (X_1_ and X_2_) for 37 runoff stations are displayed in Table 2. The production store capacity (X_1_) value varies between the allowable minimum (2.00 mm) and maximum (10.00 mm) with the average and standard deviation values of 5.71 mm and 2.49 mm, respectively. The skewness and kurtosis X_1_values of − 0.52, and − 1.03, respectively, indicated that the production store capacity (X_1_) in the southern river basin, Thailand, has left skew platykurtic, and non-symmetric distributions. The groundwater exchange rate (X_2_) value varies between 0.54 and 1.00. Most X_2_ values are 1.0, which is the maximum value, resulting in its average X2 value of 0.93 with a meagre standard deviation value of 0.12. The skewness and kurtosis values of X_2_ were − 2.01, and 3.69, respectively, indicated that the groundwater exchange rate (X_2_) in the southern river basin, Thailand, has left skew, leptokurtic, and non-symmetric distributions. It can observe that the positive obtained groundwater exchange rate (X2) value. Thus, it shows no groundwater runs out of the basin.

**Table 2:** The statistical values of the fitted GR2M model parameters.

Parameters	Min	Max	Avg	SD	SK	K
X_1_	2.00 mm	10.00 mm	5.71 mm	2.49 mm	− 0.52	− 1.03
X_2_	0.54	1.00	0.93	0.12	− 2.01	3.69

Remark: Min = Minimum; Max = Maximum; Avg = Average; SD = Standard deviation; SK = Skewness coefficient; and K = Kurtosis coefficient.

### The suitable group of independent variables for determining two GR2M parameters using regression-based methods

The results of investigating three scenarios for selecting the most suitable group of independent variables, that is, (1) scenario-1: using only the basin's physical characteristics, (2) scenario-2: using only hydrological information, and (3) scenario-3: combining those mentioned variables in scenarios 1 and 2. The results as shown in Table 3 indicates that scenario-1 received the worse performance for all cases due to giving the highest CA values than other cases. The italic number in Table 3 shows the scenario giving the best performance. For developing the regionalized X_1_ and X_2_ equations with MLR, we found that the most suitable group of independent variables was scenario-3. Also, the scenario-3 used for developing the regionalized X_1_ equation in which RF was the best independent variables. In scenario-2, the regionalized X_2_ equation developed by RF gave the best performance. By using the scenario-2, the regionalized X_1_ equation developed by M5 was the best. In scenario-3, the regionalized X_2_ equation developed by M5 gave the best performance. The explicit equations for estimating X_1_ and X_2_ values using MLR and M5 were shown in Eqs. (11) to (14). It should be noticed that the equation for X_2_ obtained from M5 method excluded variables of the basin's physical characteristics, although scenario-3 was selected the best one. RF is a machine learning algorithm and it has no an explicit equation like MLR and M5.

**Table 3:** The suitable group of independent variables for determining two GR2M parameters.

Methods	Scenario	X_1_				X_2_			
		MAE	RMSE	r	CA	MAE	RMSE	r	CA
MLR	1	2.032	2.383	0.237	1.787	0.083	0.110	0.312	0.365
	2	1.462	1.820	0.671	1.277	0.059	0.078	0.733	0.200
	3	*1.455*	*1.797*	*0.681*	*1.263*	*0.053*	*0.070*	*0.794*	*0.164*
RF	1	0.658	0.861	0.963	0.531	0.036	0.045	0.971	0.046
	2	0.595	0.790	0.976	0.478	*0.024*	*0.036*	*0.984*	*0.031*
	3	*0.580*	*0.772*	*0.974*	*0.468*	0.028	0.039	0.983	0.033
M5	1	2.072	2.453	0.000	1.842	0.083	0.109	0.312	0.365
	2	*1.226*	*1.578*	*0.790*	*1.060*	0.051	0.070	0.799	0.161
	3	1.502	1.917	0.624	1.343	*0.048*	*0.065*	*0.829*	*0.142*

#### MLR

11 $$\begin{aligned} {\text{X}}_{1} & = 3.90 \times 10^{ - 5} {\text{A}} - 1.48 \times 10^{ - 6} {\text{L}} + 2.10 \times 10^{ - 5} {\text{L}}_{{\text{c}}} - 0.109{\text{RF}}_{2} - 0.079{\text{RF}}_{3} - 0.052{\text{RF}}_{4} \\ & \quad - \;0.031{\text{RF}}_{5} - 0.073{\text{RF}}_{6} - 0.049{\text{RF}}_{7} - 0.060{\text{RF}}_{8} - 0.056{\text{RF}}_{9} - 0.039{\text{RF}}_{10} \\ & \quad - \;0.067{\text{RF}}_{11} - 0.055{\text{RF}}_{12} + 0.053{\text{RF}}_{{\text{y}}} + 7.671 \\ \end{aligned}$$

12 $$\begin{aligned} {\text{X}}_{2} & = 3.57 \times 10^{ - 5} {\text{A}} - 1.54 \times 10^{ - 6} {\text{L}} + 2.59 \times 10^{ - 7} {\text{L}}_{{\text{c}}} - 6.92 \times 10^{ - 4} {\text{RF}}_{2} - 2.07 \times 10^{ - 3} {\text{RF}}_{3} \\ & \quad - \;5.38 \times 10^{ - 4} {\text{RF}}_{4} - 1.56 \times 10^{ - 4} {\text{RF}}_{5} - 5.29 \times 10^{ - 4} {\text{RF}}_{6} - 1.19 \times 10^{ - 3} {\text{RF}}_{7} \\ & \quad - \;1.24 \times 10^{ - 3} {\text{RF}}_{8} - 1.23 \times 10^{ - 3} {\text{RF}}_{9} - 5.90 \times 10^{ - 4} {\text{RF}}_{10} - 1.68 \times 10^{ - 3} {\text{RF}}_{11} \\ & \quad - \;9.11 \times 10^{ - 4} {\text{RF}}_{12} + 7.74 \times 10^{ - 4} {\text{RF}}_{{\text{y}}} + 1.270 \\ \end{aligned}$$

#### M5

13 $$\begin{aligned} {\text{X}}_{1} & = - 0.022{\text{RF}}_{2} - 0.0012{\text{RF}}_{3} - 0.0062{\text{RF}}_{6} - 0.0041{\text{RF}}_{9} + 0.0019{\text{RF}}_{{\text{y}}} \\ & \quad + \;6.5015:{\text{if}}\;{\text{RF}}_{6} \le 152.345,\;{\text{and}}\;{\text{RF}}_{{\text{y}}} \le 1701.835 \\ {\text{X}}_{1} & = - 0.0238{\text{RF}}_{2} - 0.0013{\text{RF}}_{3} - 0.0062{\text{RF}}_{6} - 0.0016{\text{RF}}_{7} - 0.0041{\text{RF}}_{9} \\ & \quad + 0.0019{\text{RF}}_{{\text{y}}} + 6.9793:\;{\text{if }}\;{\text{RF}}_{6} \le 152.345,\;{\text{and }}\;{\text{RF}}_{{\text{y}}} > 1701.835 \\ {\text{X}}_{1} & = - 0.0549{\text{RF}}_{2} - 0.0178{\text{RF}}_{3} - 0.0048{\text{RF}}_{6} - 0.0018{\text{RF}}_{9} - 0.0065{\text{RF}}_{11} + 0.0015{\text{RF}}_{{\text{y}}} \\ & \quad + \;7.1315:{\text{if}}\;{\text{RF}}_{6} > 152.345,{\text{RF}}_{3} \le 125.635,\;{\text{and}}\;{\text{RF}}_{2} \le 36.805 \\ {\text{X}}_{1} & = - 0.057{\text{RF}}_{2} - 0.0178{\text{RF}}_{3} - 0.0048{\text{RF}}_{6} - 0.0017{\text{RF}}_{9} - 0.0071{\text{RF}}_{11} + 0.0015{\text{RF}}_{{\text{y}}} \\ & \quad + \;6.6992:{\text{if}}\;{\text{RF}}_{6} > 152.345,{\text{RF}}_{3} \le 125.635,\;{\text{and}}\;{\text{RF}}_{2} > 36.805 \\ {\text{X}}_{1} & = - 0.0368{\text{RF}}_{2} - 0.0213{\text{RF}}_{3} - 0.0048{\text{RF}}_{6} - 0.0032{\text{RF}}_{9} + 0.0015{\text{RF}}_{{\text{y}}} \\ & \quad + \;7.2107:{\text{if}}\;{\text{RF}}_{6} > 152.345,\;{\text{and}}\;{\text{RF}}_{3} > 125.635 \\ \end{aligned}$$

14 $$\begin{aligned} {\text{X}}_{2} & = - 0.0003{\text{RF}}_{8} - 0.0002{\text{RF}}_{11} + 1.0979:{\text{if RF}}_{8} \le 105.745 \\ {\text{X}}_{2} & = 0.0006{\text{RF}}_{6} - 0.0002{\text{RF}}_{8} - 0.0007{\text{RF}}_{11} + 0.0003{\text{RF}}_{12} - 0.0002{\text{RF}}_{{\text{y}}} \\ & \quad + \;1.2801:{\text{if RF}}_{8} > 105.745 \\ \end{aligned}$$

where A = basin area, L = river length, L_c_ = river length from the basin’s centroid to the basin outlet, RF_1,_ RF_2_, RF_3_, …, and RF_12_ = average monthly rainfall in January, February, March, …, and December, respectively, and RF_y_ = average annual rainfall.

### Model performance comparison in estimating the regional GR2M model parameters

In this section, the application of five methods (i.e., MLR, RF, M5, SPA, and PSA) were applied for developing the regionalized GR2M parameters, which are presented and discussed. The first three methods are based on the regression-based method and the rest two methods are distance-based method. Comparison of the fitted X_1_ and X_2_ parameters in the GR2M model and the two parameters obtained from those five methods was conducted. The results of applying those five methods to estimate X_1_ and X_2_ values are summarized in Table 4 for all 37 runoff stations. It indicated RF gave the best performance for estimating X_1_ due to providing the lowest CA value, following by MLR, M5, SPA, and PSA, respectively. Likewise, RF gave the best performance for estimating X_2_, followed by M5, MLR, SPA, and PSA.

**Table 4:** Statistical indices for estimating X_1_ and X_2_ values.

Statistical indices	X_1_					X_2_				
	MLR	RF	M5	SPA	PSA	MLR	RF	M5	SPA	PSA
MAE	1.45	0.58	1.50	1.67	1.95	0.05	0.03	0.05	0.08	0.10
RMSE	1.80	0.77	1.92	2.41	2.46	0.07	0.04	0.07	0.12	0.14
r	0.68	0.97	0.62	0.39	0.30	0.79	0.98	0.83	0.27	− 0.16
CA	1.26	0.47	1.34	1.64	1.77	0.16	0.03	0.14	0.37	0.41

### Evaluation of the regionalized parameters applied in the GR2M model

**Figure 7:**
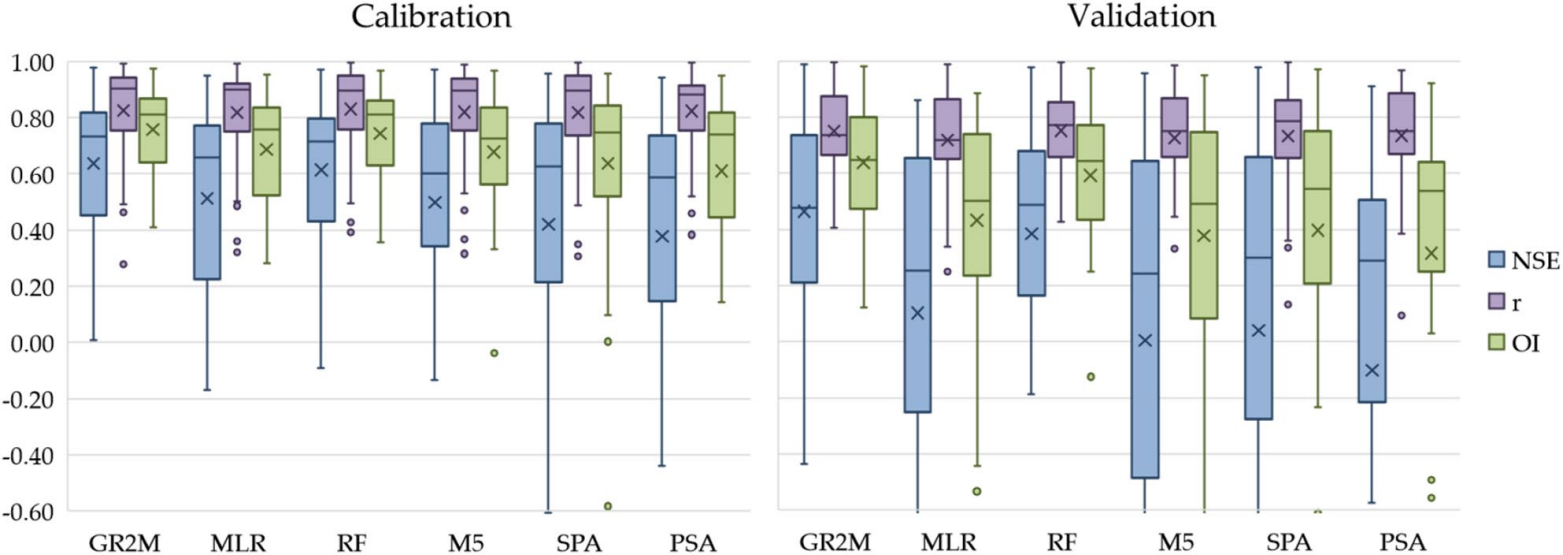
Comparative results of applying the estimated X1 and X2 values with five methods in the GR2M model.

**Figure 8:**
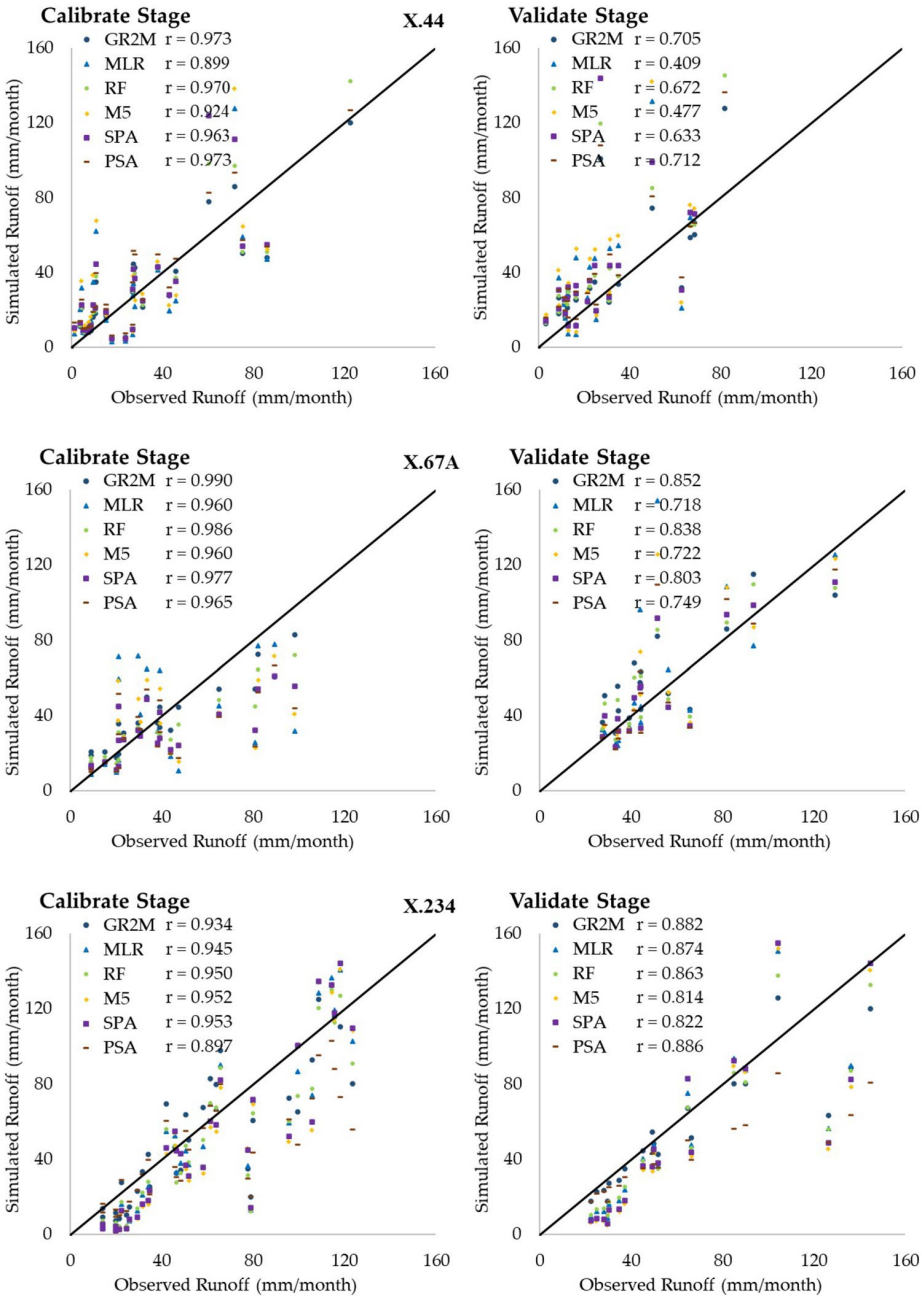
The relationship between the observed runoff and simulated runoff obtained from six methods in X.44, X.67, and X.234.

This section aims to present the performance evaluation of the regionalized GR2M parameters developed in the previous section. Those parameters, areal monthly rainfall and evapotranspiration were used as input parameters for the GR2M model. With the same input data sets of areal monthly rainfall, evapotranspiration and different X_1_ and X_2_ values obtained from five different methods, we got five monthly runoff time-series as the GR2M model’s output data. Those monthly runoff time-series were compared to that of the calibrated and validated GR2M model. Figure 7 shows the box plot graph, which was obtained from evaluating the GR2M model's effectiveness by using the regionalized GR2M parameters. Also, Table 5 presents the comparison of efficiency criteria obtained from applying X_1_ and X_2_ values in the GR2M model with five regionalized methods. As usual, the calibrated model’s performance for all methods was better than those of the validated ones. Figure 7 and Table 5 show that the average values of NSE, r, and OI obtained from the calibration stage gave better values than those obtained from the validation stage. In addition, RF gave the best results when considering NSE and OI values for both calibration and validation stages. Table 6 shows the number of runoff stations that were categorized into four groups with the same interval for each statistical index. By this way, we can see and compare the five methods' effectiveness in our experiment easily. Considering NSE, r, and OI values of equal or more than 0.70 simultaneously, we found that RF gave the best performance in monthly runoff estimation due to providing the highest total number of runoff stations of 60 (i.e., NSE, r, and OI values are equal or more than 0.70 simultaneously in those 60 stations), followed by SPA (53 stations), M5 (49 stations), MLR (46 stations), and PSA (42 stations). Figure 8 presents the scatter plot of three examples of X.44, X.67A, and X.234 runoff station. The graph shows the relationship between the observed and the simulated runoff obtained from GR2M model, MLR, RF, M5, SPA, and PSA in both calibration and validation stages. The perfect line is depicted as the 45-degree diagonal solid line.

**﻿Table 5:** The performance comparison of applying the estimated X_1_ and X_2_ values in the GR2M model with 6 methods.

Method	Efficiency criteria					
	Calibration			Validation		
	NSE	r	OI	NSE	r	OI
**GR2M**						
MAX	0.978	0.993	0.974	0.987	0.996	0.980
MIN	0.007	0.277	0.411	− 0.437	0.407	0.120
Avg	0.637	0.825	0.757	0.465	0.750	0.639
SD	0.256	0.170	0.153	0.354	0.166	0.213
**MLR**						
MAX	0.950	0.991	0.954	0.860	0.987	0.886
MIN	− 0.170	0.320	0.280	− 1.906	0.249	− 0.628
Avg	0.513	0.817	0.686	0.102	0.719	0.433
SD	0.325	0.171	0.188	0.713	0.188	0.399
**RF**						
MAX	0.970	0.995	0.968	0.979	0.995	0.972
MIN	− 0.091	0.393	0.357	− 0.881	0.428	− 0.125
Avg	0.613	0.830	0.744	0.384	0.752	0.592
SD	0.275	0.164	0.164	0.442	0.162	0.260
**M5**						
MAX	0.970	0.990	0.968	0.955	0.986	0.948
MIN	− 0.748	0.315	− 0.039	− 2.235	0.235	− 0.804
Avg	0.497	0.820	0.678	0.004	0.726	0.378
SD	0.369	0.172	0.211	0.806	0.196	0.448
**SPA**						
MAX	0.955	0.994	0.958	0.977	0.995	0.970
MIN	− 1.819	0.307	− 0.583	− 3.379	0.133	− 1.534
Avg	0.420	0.818	0.636	0.041	0.733	0.398
SD	0.580	0.179	0.323	0.902	0.194	0.512
**PSA**						
MAX	0.941	0.994	0.948	0.909	0.967	0.920
MIN	− 2.084	0.383	− 0.724	− 3.459	0.094	− 1.577
Avg	0.377	0.822	0.609	− 0.102	0.735	0.316
SD	0.567	0.156	0.320	1.035	0.194	0.577

Remark: MAX = Maximum; MIN = Minimum; Avg = Average; SD = Standard deviation.

**Table 6:** The performance criteria of the analysis method for estimating parameters.

Range	Regionalization methods (station)				
	MLR	RF	M5	SPA	PSA
0.7 ≤ NSE	9	13	8	10	2
0.5 ≤ NSE < 0.7	5	7	7	8	12
0.3 ≤ NSE < 0.5	10	9	8	5	7
NSE < 0.3	13	8	14	14	16
0.7 ≤ r	24	27	26	27	27
0.5 ≤ r < 0.7	10	9	9	6	8
0.3 ≤ r < 0.5	3	1	2	4	1
r < 0.3	0	0	0	0	1
0.7 OI	13	20	15	16	13
0.5 OI < 0.7	13	10	9	9	9
0.3 OI < 0.5	5	6	5	5	7
OI < 0.3	6	1	8	7	8

**Figure 9:**
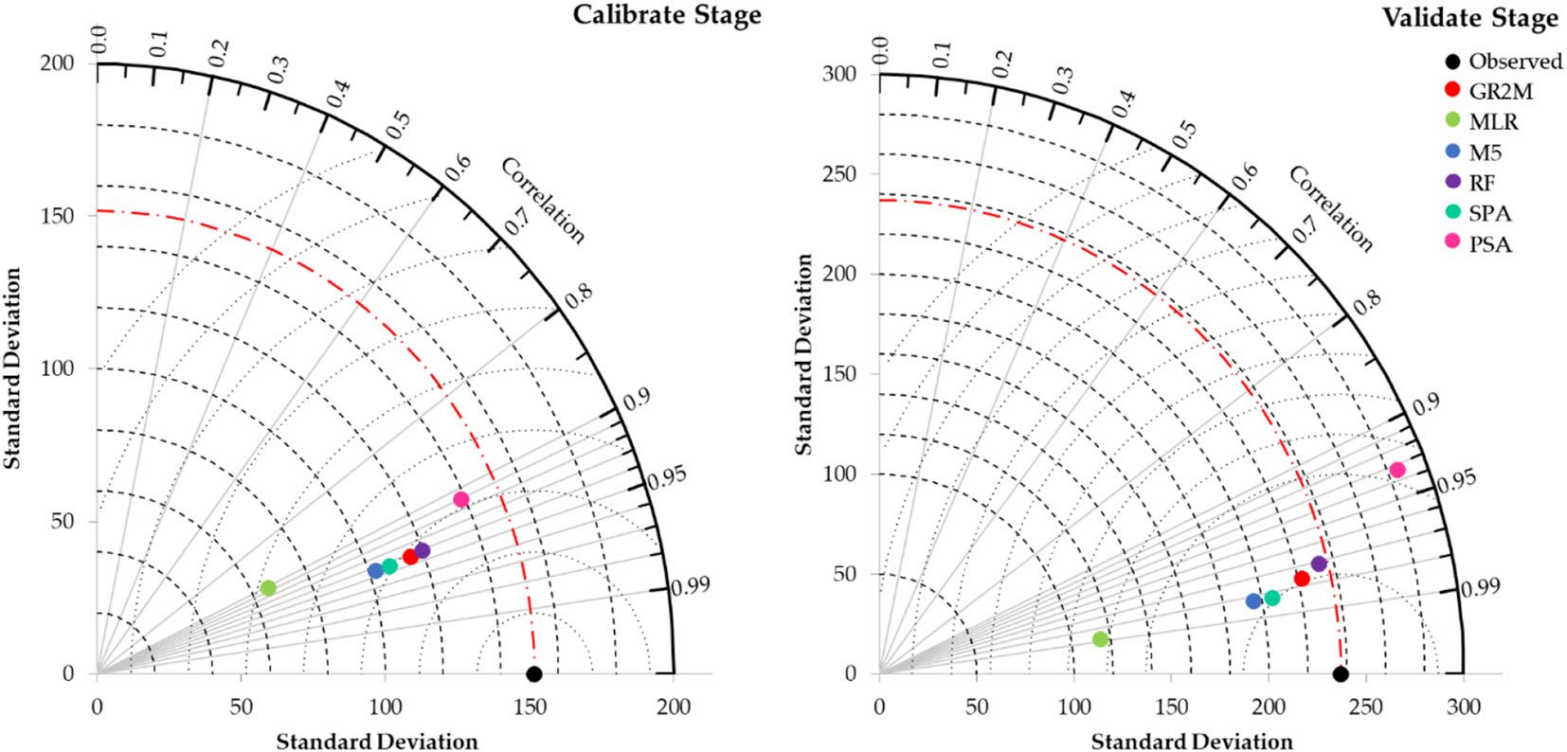
Taylor diagram for the regionalized GR2M model for training and testing stages.

Figure 9 presents a Taylor diagram that compares among five regionalized GR2M model and the calibrated and validated GR2M model. As shown in Fig. 9, all models gave a standard deviation value less than that of the observed runoff time series, except for PSA. RF provided the results closest to GR2M's results, followed by SPA, M5, PSA, and MLP. However, in case of lack of basin's physical characteristics and hydrological data, it would recommend using SPA since it only needs information on the distance between a gauged station (or donor station) and ungauged stations (or a target station).

## Conclusion

The performance investigation of the regionalized GR2M model parameters for estimating monthly runoff in the ungauged basin was conducted in this research work. We selected 37 runoff gauged stations located in the southern basin, Thailand, as the study case. The regression-based and distance-based methods were applied for this purpose. Using regression-based methods to determine two GR2M parameters, the hydrological data was more suitable group of independent variables than the basin’s physical characteristics. We also found that RF gave the best performance for estimating X_1_ and X_2_ values due to providing the lowest error, followed by M5, MLR, SPA, and PSA. However, by simultaneously considering NSE, r, and OI values, RF provided the best performance in estimating monthly runoff time series by giving NSE, r, and OI values of equal or more than 0.70, followed by SPA, M5, MLR, and PSA. Furthermore, by using a Taylor diagram, we found that RF provided the results closest to GR2M's results, followed by SPA, M5, PSA, and MLP. However, in case of lack of basin's physical characteristics and hydrological information, it would recommend using SPA since it only needs information on the distance between a gauged station (or donor station) and ungauged stations (or a target station). Estimating monthly runoff time series in the ungauged basin via the regionalization methods could be drastically useful for water resources planning and management.
